# Pancreatic Pseudocyst Hemorrhage Complicated by an Acute ST-Elevation Myocardial Infarction

**DOI:** 10.7759/cureus.13585

**Published:** 2021-02-27

**Authors:** Kevin Groudan, Kartikeya Tripathi, Kamesh Gupta, Rinad Tabbalat

**Affiliations:** 1 Internal Medicine, Baystate Medical Center, Springfield, USA; 2 Gastroenterology, Baystate Medical Center, Springfield, USA

**Keywords:** st-elevation myocardial infarction (stemi), upper gi bleed, pancreatic pseudocyst hemorrhage

## Abstract

ST-segment elevation myocardial infarction is a medical emergency that requires immediate treatment with potent anti-platelet and anti-coagulant therapies and reperfusion by percutaneous coronary intervention. The use of anti-platelet and anti-coagulant therapies can result in hemorrhagic complications, and their use is challenging in a patient with an active gastrointestinal bleed. We report the case of a patient who simultaneously presented with both an ST-segment elevation myocardial infarction and a hemorrhagic pancreatic pseudocyst. There are currently no comprehensive recommendations to guide treatment of these conditions when presenting concomitantly. This case outlines the multi-disciplinary approach taken by our cardiology and gastroenterology teams and highlights the need to develop management algorithms for these two life-threatening conditions.

## Introduction

Bleeding complication following acute coronary syndrome with the use of anti-thrombotics is associated with increased morbidity and mortality [1]. The out-of-hospital bleeding rates have doubled in the last two decades, which parallels anti-thrombotic drug use [2]. There are more than 150 million patients worldwide that are prescribed an anti-thrombotic agent [3]. Gastrointestinal (GI) bleed is associated with a four-fold increase in all-cause mortality in these cardiac patients [3]. In a patient with concomitant ST-segment elevation myocardial infarction (STEMI) and GI bleed, managing anticoagulation is a dilemma that requires balancing risks to optimize care. There are no comprehensive guidelines outlining management of these patients, and most literature on the topic is limited to case reports. We report the case of a patient admitted to the intensive care unit (ICU) for hematemesis and features of shock, likely secondary to pancreatic pseudocyst hemorrhage. He was planned for endoscopic intervention but developed an acute inferior wall STEMI, posing a management dilemma.

## Case presentation

**Figure 1:**
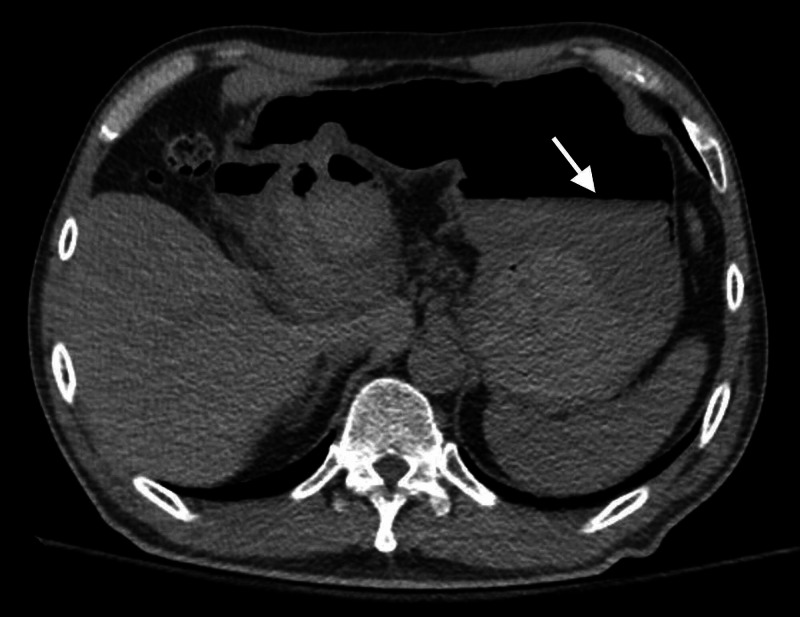
CT of the abdomen showing hemorrhage within the gastric lumen and pseudocyst

A 59-year-old man with a history significant for polycythemia vera, recurrent acute pancreatitis, status post-cholecystectomy, and recurrent pancreatic pseudocyst requiring prior stent placement presented to our hospital for large-volume hematemesis and melena after undergoing transgastric drainage of his pancreatic pseudocyst with stent placement earlier in the day. On arrival, he was hemodynamically stable with a blood pressure of 122/67 mmHg and a heart rate of 82 bpm. Admission labs were significant for a hemoglobin of 15.5 gm/dL from a baseline of 17.0 gm/dL and a troponin T of 0.01 ng/mL. He was repleted with two units of packed red blood cells in the emergency department. A computed tomography (CT) of the abdomen with angiography revealed a mixture of blood and clot within the pancreatic pseudocyst cavity with extravasation into the stomach, consistent with a pancreatic pseudocyst hemorrhage (Figure 1).

**Figure 2:**
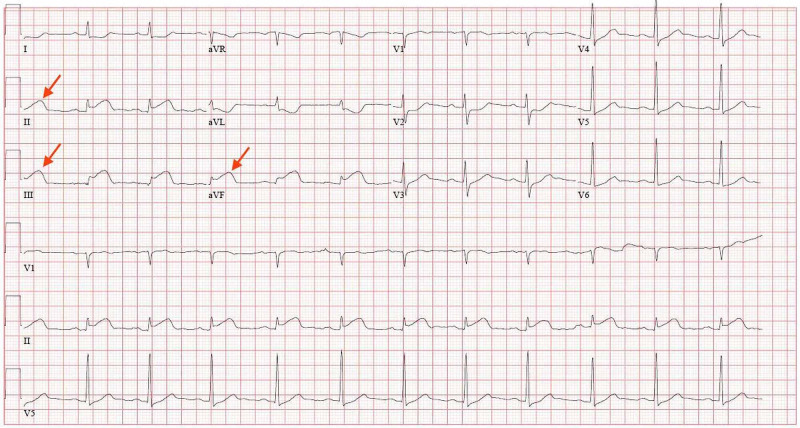
EKG showing ST-segment elevations in leads II, III, and aVF EKG, electrocardiogram

**Figure 3:**
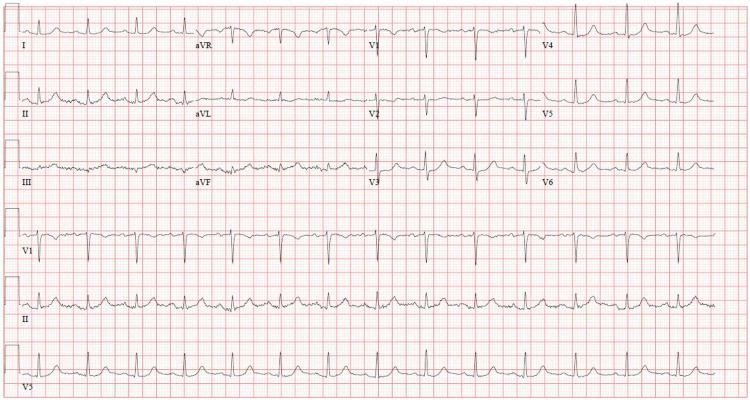
Initial EKG showing no ST-segment elevations EKG, electrocardiogram

**Figure 4:**
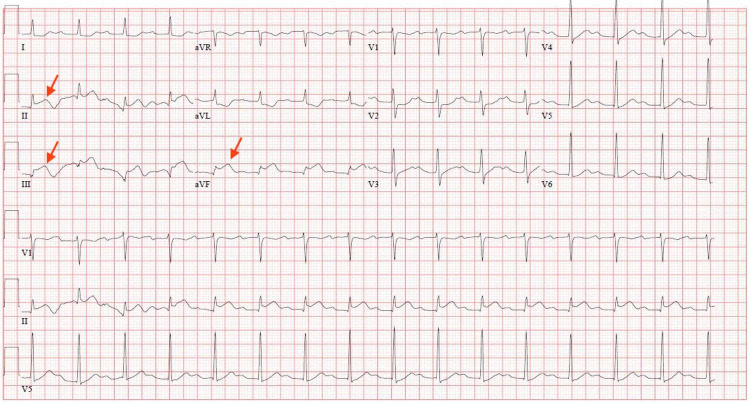
EKG showing persistent ST-segment elevations in leads II, III, and avF EKG, electrocardiogram

He was admitted to the ICU for elective intubation for urgent esophagogastroduodenoscopy; however, prior to this, he developed ventricular bigeminy on cardiac telemetry. An electrocardiogram was obtained and showed ST-segment elevations in leads II, III, and aVF (Figure 2), which were not present on electrocardiogram obtained earlier in the day (Figure 3). Shortly later, he developed acute substernal chest pain, and repeat troponin T increased from 0.01 ng/mL to 0.12 ng/mL. Findings were consistent with an inferior STEMI. Aspirin load and heparin were deferred due to his GI bleed. The patient’s chest pain subsided spontaneously; however, repeat electrocardiogram revealed persistent ST-segment elevations (Figure 4).

A collective discussion was held with the ICU, cardiology service, and gastroenterology service on how to manage his acute inferior STEMI in the context of active GI hemorrhage. The cardiology service did not recommend emergent percutaneous coronary intervention (PCI) due to the patient being free from chest pain and the increased risk of bleeding should he require stenting and dual anti-platelet therapy. The gastroenterology service recommended interventional radiology (IR) be involved in the control of pseudocyst bleeding; however, given that the patient was hemodynamically and clinically stable, it was not felt to be an emergency by the IR team.

The patient was managed conservatively with intravenous proton pump inhibitor therapy and close monitoring for recurrent bleeding or cardiac instability. Heparin infusion and dual anti-platelets were held. Over the course of three days, he remained clinically and hemodynamically stable and did not require further blood transfusions. Gastroenterology suspected his intracavitary bleed self-tamponaded and recommended outpatient follow-up with his gastroenterologist to determine removal timing of the transgastric stent. Echocardiogram showed right wall motion abnormalities in the basal inferior wall. The cardiologist started him on aspirin, atorvastatin, and metoprolol prior to discharge and scheduled an outpatient coronary CT angiogram.

## Discussion

According to the Acute Catheterization and Urgent Intervention Triage Strategy (ACUITY) trial, clinically significant GI hemorrhage may occur simultaneously in 1.3% of moderate- and high-risk cases of acute coronary syndrome [4]. This challenging scenario requires balancing of risks and benefits for the treatment of each condition. Several key aspects of our patient’s presentation were crucial to his management.

After the patient’s chest pain spontaneously resolved following his STEMI, he fortunately did not show further signs of cardiac instability, such as persistent chest pain, electrical instability, or cardiogenic shock. The presence of any of these factors would have strongly suggested that the patient was unlikely to survive without emergent PCI. The Early Revascularization in Acute Myocardial Infarction Complicated by Cardiogenic Shock (SHOCK) trial showed that emergency revascularization in patients with cardiogenic shock led to significant survival benefit after six months [5].

In the context of his hemorrhage, he demonstrated ongoing stability in hemoglobin and hematocrit (H&H) and clinical course deferring the need for urgent intervention. Important aspects of his care early in his hospital course included H&H draws every six hours and continuous telemetry for monitoring for hypotension or tachycardia. He was also monitored for further symptoms of GI blood loss, including hematemesis, hematochezia, melena, dizziness, and pallor. Although upper GI tract bleeds have a higher risk of being clinically significant, the patient’s relatively mild drop in baseline hemoglobin on arrival to the hospital and lack of bright red blood per rectum were reassuring that his upper GI bleed was not brisk [6].

## Conclusions

STEMI is a potentially life-threatening condition that requires emergent treatment with anti-platelet and anti-coagulant therapies. When STEMI occurs concomitantly with GI hemorrhage, patients should be evaluated for the risk of hemorrhagic complications from this regimen. There are currently no comprehensive recommendations guiding the management of concomitant STEMI and GI hemorrhage. This case identifies risks and strategies to manage concomitant STEMI and GI hemorrhage and highlights the need for further research on the subject.
